# Use of snake antivenom in the Region of the Americas: a systematic review

**DOI:** 10.2471/BLT.24.291941

**Published:** 2025-09-16

**Authors:** Clara Guerra-Duarte, Viviane Pauline de Sousa, Gladstony de Oliveira-Sousa, Marcos Paulo Gomes Mol

**Affiliations:** a Diretoria de Pesquisa e Desenvolvimento, Fundação Ezequiel Dias, Belo Horizonte, Minas Gerais, Brazil.; b Departamento de Bioquímica e Imunologia, Instituto de Ciências Biológicas, Universidade Federal de Minas Gerais, Belo Horizonte, Brazil.

## Abstract

**Objective:**

To evaluate the use of antivenom therapy in *Bothrops* envenomation in the Region of the Americas and to synthesize data on treatment outcomes, adverse reactions and long-term sequelae.

**Methods:**

We systematically searched Web of Science, Scopus, Lilacs, PubMed® and Google Scholar for studies published up to 5 May 2025 that reported on the effects of antivenom treatment in humans bitten by *Bothrops* species in the Region of the Americas. We extracted data on patient demographics, adverse reactions, clinical complications and long-term sequelae following antivenom therapy.

**Findings:**

Of 2060 articles identified, 38 met the inclusion criteria. *Bothrops* envenomations occurred more frequently in men (75.2%; 3247/4320 individuals), predominantly affected the lower limbs (76.5%; 2494/3295) and typically resulted in moderate-grade envenoming (44.6%; 1553/3483 individuals). We found that adverse reactions to antivenom therapy were common: 19.6% (589/2998) experienced early reactions and 1.6% (16/992) delayed reactions, although incidence declined in recent years. Individuals experienced clinical complications, such as severe oedema (23.2%; 239/1032), secondary infections (22.8%; 452/1985) and coagulopathies (20.7%; 357/1724). Some patients also experienced permanent sequelae, though these complications were relatively infrequent (3.3%; 50/1512). For studies reporting on deaths, 0.8% (23/3035) of patients died.

**Conclusion:**

Antivenom therapy remains central to the management of *Bothrops* envenomation. However, challenges persist in treatment outcomes and long-term sequelae. Addressing these challenges requires ongoing research to strengthen antivenom manufacturing, explore adjunct therapies and improve post-envenomation care. Substantial heterogeneity study methods and reported outcomes, precluded the ability to conduct pooled analyses and generalize findings.

## Résumé

**Objectif:**

Évaluer l’utilisation d’un traitement antivenimeux dans les cas d’envenimation par des serpents du genre *Bothrops* dans la région des Amériques et synthétiser les données sur les résultats du traitement, les effets indésirables et les séquelles à long terme.

**Méthodes:**

Nous avons effectué une recherche systématique dans Web of Science, Scopus, Lilacs, PubMed^®^ et Google Scholar pour trouver des études publiées jusqu’au 5 mai 2025 qui rendaient compte des effets d’un traitement antivenimeux chez les humains mordus par des espèces appartenant au genre *Bothrops* dans la région des Amériques. Nous avons extrait des données sur les caractéristiques démographiques des patients, les effets indésirables, les complications cliniques et les séquelles à long terme après un traitement antivenimeux.

**Résultats:**

Sur les 2 060 articles identifiés, 38 répondaient aux critères d’inclusion. Les envenimations par des représentants du genre *Bothrops* étaient plus fréquentes chez les hommes (75,2%; 3 247/4 320 individus), concernaient principalement les membres inférieurs (76,5%; 2 494/3 295) et entraînaient généralement une envenimation modérée (44,6%; 1 553/3 483). Nous avons constaté que les effets indésirables du traitement antivenimeux étaient fréquents: 19,6% (589/2 998) présentaient des réactions précoces et 1,6% (16/992) des réactions tardives, bien que l’incidence ait diminué ces dernières années. Des individus ont fait état de complications cliniques, telles qu’un œdème sévère (23,2%; 239/1 032), des infections secondaires (22,8%; 452/1 985) et une incoagulabilité sanguine (20,7%; 357/1 724). Certains patients ont également souffert de séquelles permanentes, bien que ces complications étaient relativement rares (3,3%; 50/1 512). Dans les études signalant des décès, 0,8% (23/3 035) des patients sont décédés.

**Conclusion:**

Un traitement antivenimeux reste essentiel dans la prise en charge des envenimations par le genre *Bothrops*. Cependant, des défis persistent en ce qui concerne les résultats du traitement et les séquelles à long terme. Pour relever ces défis, il importe de poursuivre les recherches afin de renforcer la fabrication d’antivenins, d’explorer des compléments thérapeutiques et d’améliorer les soins après l’envenimation. L’hétérogénéité importante des méthodes d’étude et des résultats rapportés empêchait de réaliser des analyses groupées et de généraliser les conclusions.

## Resumen

**Objetivo:**

Evaluar el uso de la terapia con antivenenos en el envenenamiento por *Bothrops* en la Región de las Américas y sintetizar los datos sobre resultados del tratamiento, reacciones adversas y secuelas a largo plazo.

**Métodos:**

Se realizaron búsquedas sistemáticas en Web of Science, Scopus, LILACS, PubMed® y Google Scholar de estudios publicados hasta el 5 de mayo de 2025 que informaran sobre los efectos del tratamiento con antivenenos en personas mordidas por especies de *Bothrops* en la Región de las Américas. Se extrajeron datos sobre características demográficas de los pacientes, reacciones adversas, complicaciones clínicas y secuelas a largo plazo tras la terapia con antivenenos.

**Resultados:**

De 2060 artículos identificados, 38 cumplieron los criterios de inclusión. Los envenenamientos por *Bothrops* se produjeron con mayor frecuencia en hombres (75,2%; 3247/4320 personas), afectaron predominantemente a las extremidades inferiores (76,5%; 2494/3295) y, en general, dieron lugar a envenenamientos de grado moderado (44,6%; 1553/3483 personas). Se observó que las reacciones adversas a la terapia con antivenenos eran comunes: el 19,6% (589/2998) presentó reacciones tempranas y el 1,6% (16/992) reacciones tardías, aunque la incidencia disminuyó en los últimos años. Las personas experimentaron complicaciones clínicas, como edema grave (23,2%; 239/1032), infecciones secundarias (22,8%; 452/1985) y sangre incoagulable (20,7%; 357/1724). Algunas personas también presentaron secuelas permanentes, aunque estas complicaciones fueron relativamente poco frecuentes (3,3%; 50/1512). En los estudios que informaron sobre muertes, falleció el 0,8% (23/3035) de los pacientes.

**Conclusión:**

La terapia con antivenenos sigue siendo fundamental para el tratamiento del envenenamiento por *Bothrops*. Sin embargo, persisten desafíos en cuanto a los resultados del tratamiento y las secuelas a largo plazo. Abordar estos desafíos requiere investigaciones continuas para fortalecer la producción de antivenenos, explorar terapias complementarias y mejorar la atención posterior al envenenamiento. La considerable heterogeneidad en los métodos de los estudios y en los resultados reportados impidió la realización de análisis combinados y la generalización de los hallazgos.

## ملخص

الغرض تقييم استخدام العلاج بمضادات السموم في حالات تسمم أفاعي السمام في منطقة الأمريكتين، وتجميع البيانات المتعلقة بنتائج العلاج، والآثار الجانبية، والعواقب طويلة المدى.

الطريقة قمنا بالبحث بشكل منهجي في مواقع Web of Science، وScopus، وLilacs، و®PubMed ®، وGoogle Scholar، عن الدراسات المنشورة حتى 5 مايو/أيار 2025، والتي تناولت آثار العلاج بمضادات السموم على البشر الذين لدغتهم فصائل أفاعي السمام في منطقة الأمريكتين. استخرجنا بيانات عن التركيبة السكانية للمرضى، والآثار الجانبية، والمضاعفات الإكلينيكية، والعواقب طويلة المدى التي تلي العلاج بمضادات السموم.

النتائج من بين الـ 2060 مقالة التي تم تحديدها، استوفت 38 منها معايير الاشتمال. كانت حالات تسمم أفعى الدساس أكثر شيوعًا لدى الرجال (%75.2؛ 3247/4320 فردًا)، وأصابت الأطراف السفلية بشكل رئيسي (%76.5؛ 2494/3295 فردًا)، ونتج عنها عادةً تسمم متوسط الدرجة (%44.6؛ 1553/3483 فردًا). وجدنا أن الآثار الجانبية للعلاج بمضادات السموم كانت شائعة: %19.6 (589/2998) عانوا من ردود فعل مبكرة، و%1.6 (16/992) عانوا من ردود فعل متأخرة، على الرغم من انخفاض معدل حدوثها في السنوات الأخيرة. عانى الأفراد من مضاعفات إكلينيكية، مثل الوذمة الشديدة (%23.2؛ 239/1032)، والالتهابات الثانوية (%22.8؛ 452/1985)، وعدم تخثر الدم (%20.7؛ 357/1724). عانى بعض المرضى أيضًا من مضاعفات دائمة، على الرغم من أن هذه المضاعفات كانت نادرة نسبيًا (%3.3؛ 50/1512). بالنسبة للدراسات التي أبلغت عن وفيات، توفي %0.8 (23/3035) من المرضى.

الاستنتاج لا يزال العلاج بمضادات السموم أساسيًا في التعامل مع تسمم أفعى السمام. ومع ذلك، لا تزال هناك تحديات في نتائج العلاج والعواقب طويلة المدى. تتطلب معالجة هذه التحديات بحثًا مستمرًا لتعزيز تصنيع مضادات السموم، واستكشاف العلاجات المساعدة، وتحسين رعاية ما بعد التسمم. وقد حال التباين الكبير في أساليب الدراسة والنتائج المبلغ عنها دون القدرة على إجراء تحليلات مجمعة وتعميم النتائج.

## 摘要

**目的:**

旨在评估抗蛇毒血清疗法在治疗被美洲区域矛头蝮 (*Bothrops*) 咬伤所致中毒方面的使用情况并汇总治疗效果、不良反应和长期后遗症相关数据。

**方法:**

我们系统地搜索了 Web of Science、Scopus、Lilacs、PubMed® 和谷歌学术搜索引擎,以查找在 2025 年 5 月 5 日之前发表的汇报了治疗被美洲区域矛头蝮 (*Bothrops*) 咬伤的人类时所采用抗蛇毒血清的治疗效果的研究报告。我们提取了接受抗蛇毒血清治疗的患者的人口统计信息及其之后的不良反应、临床并发症和长期后遗症相关数据。

**结果:**

在我们确定的 2,060 篇文章中,有 38 篇符合纳入标准。因被矛头蝮 (*Bothrops*) 咬伤而导致中毒的患者以男性居多(75.2%,3,247 例/4320 例),绝大多数患者的被咬伤部位位于下肢(76.5%,2,494/3,295),且此类患者通常会出现中度中毒症状(44.6%,1,553 例/3,483 例)。我们发现,接受抗蛇毒血清治疗的患者出现不良反应的情况比较普遍:19.6% (589/2,998) 的患者出现了早期不良反应,而 1.6% (16/992) 的患者则出现了迟发性不良反应,但是最近几年内不良反应的发生率呈下降趋势。有些患者出现了临床并发症,例如严重水肿(23.2%,239/1,032)、继发性感染(22.8%,452/1,985)和血液无法凝固(20.7%,357/1,724)。有些患者还出现了永久性后遗症,不过出现此类并发症的患者相对较少(3.3%,50/1,512)。在汇报了死亡人数的研究报告中,患者死亡率为 0.8% (23/3,035)。

**结论:**

抗蛇毒血清疗法仍然是治疗矛头蝮 (*Bothrops*) 咬伤所致中毒的最重要手段。然而,目前仍然难以保证治疗效果并减少长期后遗症。如要解决此类难题,需要持续开展研究以加快抗蛇毒血清生产速度、探索辅助疗法并改善中毒后护理。研究方法和汇报的治疗效果之间存在显著异质性,这导致我们无法开展汇集分析和概括研究结果。

## Резюме

**Цель:**

Оценить использование антитоксинов против яда змей рода *Bothrops* в Американском регионе и свести воедино данные о результатах лечения, неблагоприятных реакциях и отдаленных последствиях.

**Методы:**

Авторы провели систематический поиск по базам данных Web of Science, Scopus, Lilacs, PubMed® и Google Scholar исследований, опубликованных до 5 мая 2025 года, в которых сообщалось об эффектах применения антитоксинов у людей, укушенных змеями рода *Bothrops*, в Американском регионе. Из исследований были извлечены данные демографического характера, сообщения о неблагоприятных реакциях, клинических осложнениях и долгосрочных последствиях применения антитоксинов.

**Результаты:**

Из 2060 полученных статей об исследованиях 38 соответствовали критериям включения. Змеи рода *Bothrops* чаще кусали мужчин (75,2%, 3247/4320 лиц), преимущественно страдали нижние конечности (76,5%, 2494/3295), и, как правило, отравление было умеренно выраженным (44,6%, 1553/3483 лиц). Авторы выявили, что неблагоприятные реакции на антитоксин случаются часто: 19,6% (589/2998) человек имели раннюю реакцию, 1,6% (16/992) – отложенную во времени, хотя в последние годы частота таких реакций сократилась. У пострадавших наблюдались клинические осложнения, такие как тяжелый отек (23,2%, 239/1032), вторичные инфекции (22,8%, 452/1985) и несвертываемость крови (20,7%, 357/1724). У некоторых пациентов последствия были необратимыми, хотя такие осложнения встречались сравнительно нечасто (3,3%, 50/1512). По данным исследований, сообщавших о смерти, летальный исход наблюдался у 0,8% (23/3035) пациентов.

**Вывод:**

Применение антитоксина остается центральным методом лечения при укусе человека змеей рода *Bothrops*. Однако и в результатах лечения, и в отдаленных последствиях наблюдаются устойчивые проблемы. Для их решения необходимы постоянные исследования, направленные на усовершенствование производства антитоксинов, изучение дополнительных методов терапии и улучшение ухода за пострадавшими от змеиного укуса. Из-за существенной разнородности методов исследования и сообщенных исходов авторам не удалось провести обобщенный анализ и сформулировать выводы общего характера.

## Introduction

Antivenom therapy, developed over a century ago, remains the main treatment for snakebite.^1^ However, clinical trials under contemporary standards to ascertain its efficacy and safety were not conducted during its development.^2^ Today, producers do quality control assessments of antivenoms in animal models, primarily focusing on the antivenoms’ ability to neutralize lethal effects,^3^ while disregarding other effects that can contribute to morbidity, such as adverse reactions and prevention of local damage.^4^^–^^6^ The few well-designed clinical trials that have been conducted on human snakebite treatment with antivenoms,^7^ consistently indicate a benefit from using antivenoms.^8^^–^^15^

In the Region of the Americas, vipers from the *Bothrops* genus are the primary cause of snakebites. This genus comprises 47 species, and is found from Mexico to Argentina, including the Caribbean and smaller Atlantic coastal islands of Brazil.^16^ Envenomation by *Bothrops* snakes triggers a range of haemotoxic effects, typically beginning with local bleeding, and may progress to include symptoms such as oedema, pain, bruising and blisters. Severe complications can arise, including haemorrhagic manifestations, worsening of local effects, infections, necrosis and compartment syndrome caused by increasing oedema. Systemic complications such as acute kidney injury, central nervous system bleeding, shock or sepsis may also occur. Ultimately, *Bothrops* envenomation can result in tissue loss, motor deficits, amputation, renal failure and death.^17^

Compared to other snakebite hotspot areas, Latin America has lower mortality, around 0.04 deaths per 100 000 population.^18^ In contrast, a systematic review showed higher mortality on the continents of Africa and Asia, at 0.44 and 0.96 deaths per 100 000 population, respectively.^19^ A reason for this difference can be the accessibility to high-quality antivenom producers in the Region of the Americas.^20^ Despite the availability, several challenges related to snakebite treatment persist in Latin America. These include critical gaps in antivenom distribution and cold-chain logistics that delay on-time administration; the mismatch between antivenom specificity and the high regional diversity of venomous species; and variations in the neutralizing potency of available antivenoms towards the different venom effects. 

To comprehensively analyse the use of antivenom therapy against *Bothrops* envenomation in the Region of the Americas, we conducted a systematic review to estimate the local and systemic short- and long-term effects in patients receiving antivenom therapy.

## Methods

We registered the systematic review in PROSPERO (CRD42020205978), in accordance with the PRISMA 2020 statement.^21^

### Search and data extraction

Up to 5 May 2025, we searched Web of Science, Scopus, Lilacs, PubMed® and Google Scholar using the search string *Bothrops AND (antivenom OR immunotherapy) AND (efficacy OR effectiveness)* to identify studies in any language, conducted in any country or territory in the Region of the Americas that reported on the effects of antivenom in human patients. We also manually examined the bibliographic references of the included articles as well as non-indexed literature.

Two reviewers independently screened the titles of articles, followed by the evaluation of abstracts and complete reading of the article. Agreement between the reviewers was needed for inclusion of a paper. If consensus was not reached, a third reviewer resolved the disagreement. Articles not specifying the snake *Bothrops sp.* as the cause of the snakebite, or did not mention a country or territory in the Region of the Americas as the study location were excluded, as well as systematic reviews, articles without numerical data from clinical studies, and animal studies. We recorded the identified articles in an Excel spreadsheet (Microsoft, Redmond, United States of America).^22^

From the included articles, we extracted study type; number of patients treated; sex; age; snakebite data; antivenom used; antivenom dosage; case evolution; and country or territory where the incident occurred. We also extracted data on variables related to post-antivenom symptoms. As noted in a previous study,^7^ snakebite clinical trials often exhibit significant heterogeneity in outcome measurements. Therefore, we focused on the relevant symptoms persisting after antivenom therapy more commonly reported across the articles. Selected variables included: bleeding (systemic or local); coagulopathies (after 6 hours of serotherapy); local damage (such as blisters, necrosis or gangrene); severe oedema or compartment syndrome; secondary infection or abscess; and acute kidney injury. We also recorded adverse reactions to antivenom, as well as other outcomes following treatment such as death, permanent sequelae, need for haemodialysis and length of hospital stay.

### Quality

We evaluated the quality of the included studies using an adapted version of a critical appraisal guideline for health research literature,^23^ which is suitable for prevalence studies. The guideline considers eight criteria, with each fulfilled criterion receiving one point. We classified studies scoring fewer than three points as low quality; those scoring between 3 and 6 points as sufficient quality; and those scoring 7 or 8 points as high quality. No studies were excluded based on methodological quality.

## Results

**Fig. 1:**
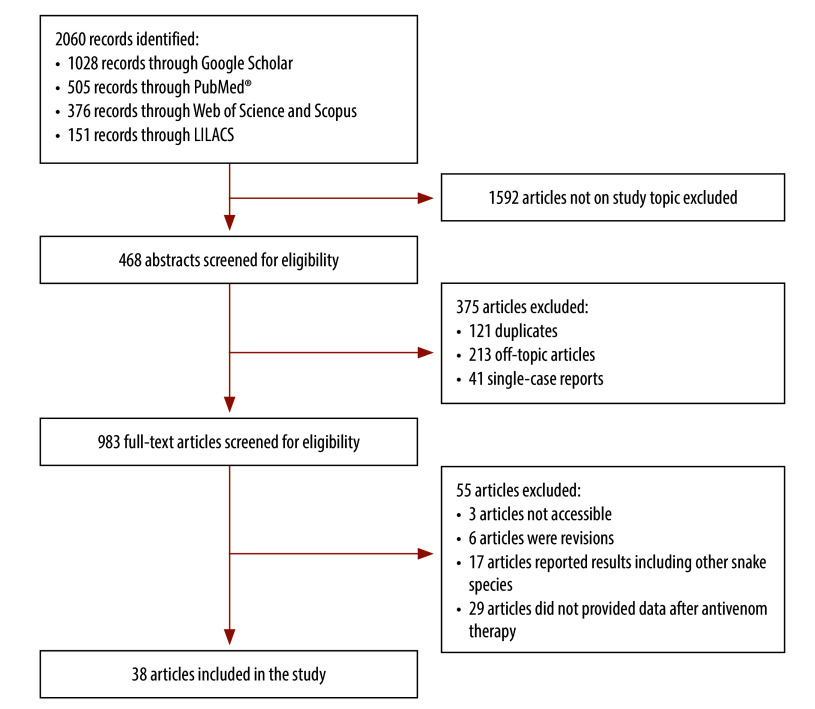
Flowchart of the selection of studies on the use of snake antivenom in the Region of the Americas

The literature search yielded a total of 2060 articles related to antivenom use in *Bothrops* envenomation. Among these, 1592 articles were off-topic and excluded. We screened 468 abstracts and included 93 articles for full-text assessment, of which 38 met the inclusion criteria (Fig. 1).^9^^–^^15^^,^^24^^–^^54^

Of the included studies, 16 were randomized controlled clinical trials,^9^^–^^15^^,^^28^^,^^32^^,^^34^^,^^35^^,^^37^^–^^39^^,^^41^^,^^42^ 14 were prospective studies^24^^–^^27^^,^^31^^,^^33^^,^^40^^,45,^^47^^–^^51^^,^^54^ and eight were retrospective studies.^29^^,^^30^^,^^36^^,^^43^^,^^44^^,^^46^^,^^52^^,^^53^ While *Bothrops* bites occur across the Region of the Americas, we only identified studies from a handful of countries or territories that have conducted studies on antivenom treatment: Brazil (20 studies);^12^^,^^13^^,^^15^^,^^26^^,^^27^^,^^30^^,^^32^^,^^33^^,^^35^^,^^36^^,^^40^^–^^43^^,45,^^47^^–^^49^^,^^51^^,^^52^ Colombia (seven studies);^9^^,^^10^^,^^14^^,^^28^^,^^34^^,^^37^^,^^39^ French Guiana (four studies);^24^^,^^44^^,^^50^^,^^54^ Martinique (three studies);^29^^,^^31^^,^^53^ Ecuador (two studies);^11^^,^^38^ Costa Rica (one study);^25^ and Uruguay (one study).^46^ The included studies were published between 1985 and 2025, with frequent publications in the late 1990s and early 2000s, and a resurgence from 2017 onwards, with 39.5% (15/38) published since 2017 (Table 1).

**Table 1:** Description of included studies on the use of snake antivenom in the Region of the Americas

Study	Study type	Country or territory	Sample size	Age range of study population, years	Study period
Barrantes et al., 1985^25^	Prospective	Costa Rica	13	NR	1985
Kouyoumdjian et al., 1988^26^	Prospective	Brazil	37	20–83	1982–1987
Kouyoumdjian et al., 1989^27^	Prospective	Brazil	22	1–69	1986–1987
Cardoso et al., 1993^13^	Randomized clinical trial	Brazil	121	7–68	1989–1991
Jorge et al., 1995^12^	Randomized clinical trial	Brazil	170	9–66	1988–1991
Otero et al., 1996^28^	Randomized clinical trial	Colombia	39	NR	1994–1996
Bucher et al., 1997^29^	Retrospective	Martinique	40	8–82	1993–1995
Milani Junior et al., 1997^30^	Retrospective	Brazil	29	NR	1975–1995
Otero-Patiño et al., 1998^10^	Randomized clinical trial	Colombia	79	NR	1994–1996
Thomas et al., 1998^31^	Prospective	Martinique	68	6–82	1993–1997
Fan et al., 1999^32^	Randomized clinical trial	Brazil	101	NR	1994–1995
Otero et al., 1999^9^	Randomized clinical trial	Colombia	53	NR	1996–1997
Bucaretchi et al., 2001^33^	Prospective	Brazil	73	1–14	1984–1999
Otero et al., 2002^34^	Randomized clinical trial	Colombia	39	NR	1999–2000
Pardal et al., 2004^15^	Randomized clinical trial	Brazil	74	5–68	1998–2000
Jorge et al., 2004^35^	Randomized clinical trial	Brazil	251	NR	1990–1996
Smalligan et al., 2004^11^	Randomized clinical trial	Ecuador	210	2–80	1997–2001
Otero et al., 2006^14^	Randomized clinical trial	Colombia	67	NR	2003–2004
Mise et al., 2007^36^	Retrospective	Brazil	665	NR	2001
Otero-Patiño et al., 2007^37^	Randomized clinical trial	Colombia	53	NR	2003
Caron et al., 2009^38^	Randomized clinical trial	Ecuador	129	1–70	1997–2001 and 2004–2006
Otero-Patiño et al., 2012^39^	Randomized clinical trial	Colombia	72	NR	2006–2008
da Silva & Tavares, 2012^40^	Prospective	Brazil	102	12–70	NR
Mendonça-da-Silva et al., 2017^41^	Randomized clinical trial	Brazil	44	> 12	2005–2008
Sachett et al., 2017^42^	Randomized clinical trial	Brazil	186	NR	2014–2016
Oliveira et al., 2019^43^	Retrospective	Brazil	412	NR	2013–2016
Resiére et al., 2020^24^	Prospective	French Guyana	83	29–52	2016–2019
Silva-de-Oliveira et al., 2020^44^	Prospective	Brazil	100	14–79	2016–2017
Heckmann et al., 2021^45^	Retrospective	French Guyana	42	16–44	2014–2017
Negrin et al., 2021^46^	Retrospective	Uruguay	49	NR	2018
Gimenes et al., 2021^47^	Prospective	Brazil	5	NR	NR
Brasileiro-Martins et al., 2022^48^	Prospective	Brazil	127	0–60	2019–2020
Soares et al., 2022^49^	Prospective	Brazil	186	NR	2014–2016
Houcke et al., 2023^50^	Prospective	French Guyana	119	Mean: 41	2016–2022
Toffano et al. 2023^51^	Prospective	Brazil	47	Mean:45	2018–2019
Coutinho et al., 2023^52^	Retrospective	Brazil	268	1–93	2018–2019
Resiére et al., 2024^53^	Retrospective	Martinique	389	Mean:46	2000–2023
Pujo et al., 2025^54^	Prospective	French Guyana	101	Mean:43	2016–2023

NR: not reported.

The included studies encompassed data from 4665 victims aged 1 to 93 years (Table 1). Men constituted most of the victims (75.2%; 3247/4320 individuals). The most commonly reported *Bothrops* species involved were *Bothrops asper* (seven studies) ^10^^,^^14^^,^^25^^,^^28^^,^^34^^,^^37^^,^^39^ and *Bothrops atrox* (eight studies;^11^^,^^15^^,^^37^^,^^38^^,^^44^^,^^47^^,^^48^^,^^54^ Table 2).

**Table 2:** Description of envenoming characteristics for each included study from the Region of the Americas

Study	*Bothrops* species	Sex of bite victim, no. (%)			Bite site, no.			Envenoming grade, no.				Received lay first aid interventions,^a^ no. (%)	Mean time to treatment, hours		Antivenom		
		Male	Female		Inferior limb	Superior limb		Mild	Moderate	Severe					Manufacturer	Molecule	Mean no. of 10mL doses
Barrantes et al., 1985^25^	*asper*	NR	NR		NR	NR		4 (33.3)	3 (25.0)	5 (41.7)		NR	NR		Instituto Clodomiro Picado	IgG	1.5
Kouyoumdjian et al., 1988^26^	*moojeni*	28 (75.7)	9 (24.3)		27 (73.0)	10 (27.0)		9 (25.0)	22 (61.1)	5 (13.9)		35 (94.6)	3.4		Brazilian health ministry accredited producers	F(ab’)2	6.0
Kouyoumdjian et al., 1989^27^	*moojeni*	18 (81.8)	4 (18.2)		18 (81.8)	4 (18.2)		5 (23.8)	13 (61.9)	3 (14.3)		NR	4.1		Brazilian health ministry accredited producers	F(ab’)2	6.2
Cardoso et al., 1993^13^	*jararaca*	89 (73.6)	32 (26.4)		98 (81.0)	23 (19.0)		0 (0.0)	89 (73.6)	32 (26.4)		65 (53.7)	4.9		Brazilian health ministry accredited producers	F(ab’)2	5.5
Jorge et al., 1995^12^	*jararaca*	137 (80.6)	33 (19.4)		137 (80.6)	33 (19.4)		NR	NR	NR		82 (48.2)	4.6		Brazilian health ministry accredited producers	F(ab’)2	3.5
Otero et al., 1996^28^	*asper*	NR	NR		NR	NR		15 (38.5)	15 (38.5)	9 (23.1)		26 (66.6)	10.9		Instituto Clodomiro Picado	IgG	NR
Bucher et al., 1997^29^	*lanceolatus*	30 (75.0)	10 (25.0)		23 (57.5)	17 (42.5)		20 (50.0)	17 (42.5)	3 (7.5)		NR	1.5		Institut Pasteur	F(ab’)2	3.0
Milani Junior et al., 1997^30^	*jararacussu*	19 (65.5)	10 (34.5)		21 (72.4)	8 (27.6)		NR	NR	NR		NR	8.2		Brazilian health ministry accredited producers	F(ab’)2	NR
Otero-Patiño et al., 1998^10^	*asper*	NR	NR		NR	NR		33 (41.8)	22 (27.8)	24 (30.4)		58 (73.4)	8.5		Brazilian health ministry accredited producers; Instituto Nacional de Salud Colombia	IgG and/or F(ab’)2	NR
Thomas et al., 1998^31^	*lanceolatus*	53 (77.9)	15 (22.1)		41 (60.3)	27 (39.7)		41 (63.1)	20 (30.8)	4 (6.2)		NR	4.5		Institut Pasteur	F(ab’)2	3.0
Fan et al., 1999^32^	*sp.*	80 (79.2)	21 (20.8)		NR	NR		76 (75.2)	25 (24.8)	0 (0.0)		NR	NR		Brazilian health ministry accredited producers	F(ab’)2	NR
Otero et al., 1999^9^	*sp.*	NR	NR		NR	NR		NR	NR	NR		NR	NR		Instituto Clodomiro Picado	IgG	NR
Bucaretchi et al., 2001^33^	*jararaca*	48 (65.8)	25 (34.2)		62 (84.9)	11 (15.1)		19 (26.8)	37 (52.1)	15 (21.1)		13 (17.8)	3.2		Brazilian health ministry accredited producers	F(ab’)2	6.0
Otero et al., 2002^34^	*asper*	31 (79.5)	8 (20.5)		23 (59.0)	16 (41.0)		2 (5.1)	8 (20.5)	29 (74.4)		21 (53.8)	NR		Probiol; Instituto Nacional de Salud Colombia; Bioclon	IgG and/or F(ab')2	9.1
Pardal et al., 2004^15^	*atrox*	64 (86.5)	10 (13.5)		65 (87.8)	9 (12.2)		43 (58.1)	27 (36.5)	4 (5.4)		28 (37.8)	NR		Brazilian health ministry accredited producers	F(ab’)2	5.8
Jorge et al., 2004^35^	*sp.*	204 (81.3)	47 (18.7)		NR	NR		NR	NR	NR		126 (50.2)	4.2		Brazilian health ministry accredited producers	F(ab’)2	NR
Smalligan et al., 2004^11^	*atrox*	106 (50.5)	104 (49.5)		101 (52.9)	90 (47.1)		NR	NR	NR		NR	7.8		Brazilian health ministry accredited producers; Instituto Nacional de Salud Colombia; Instituto Nacional de Higiene y Medicina Tropical Leopoldo Izquieta Pérez	IgG and/or F(ab')2	NR
Otero et al., 2006^14^	*asper*	47 (70.1)	20 (29.9)		54 (80.6)	13 (19.4)		17 (25.4)	35 (52.2)	15 (22.4)		32 (47.7)	7.9		Instituto Clodomiro Picado	IgG	NR
Mise et al., 2007^36^	*sp.*	510 (76.7)	155 (23.3)		500 (75.2)	165 (24.8)		127 (21.1)	318 (52.8)	157 (26.1)		70 (10.5)	NR		Brazilian health ministry accredited producers	F(ab’)2	7.7
Otero-Patiño et al., 2007^37^	*asper and atrox*	37 (69.8)	16 (30.2)		38 (71.7)	15 (28.3)		13 (24.5)	30 (56.6)	10 (18.9)		23 (44.2)	NR		Bioclon	F(ab’)2	5.5
Caron et al., 2009^38^	*atrox and bilineatus*	67 (51.9)	62 (48.1)		NR	NR		NR	NR	NR		NR	5.2		Brazilian health ministry accredited producers	F(ab’)2	2.0
Otero-Patiño et al., 2012^39^	*asper*	48 (66.7)	24 (33.3)		61 (84.7)	11 (15.3)		19 (26.4)	36 (50.0)	17 (23.6)		27 (37.5)	5.4		Instituto Clodomiro Picado	IgG and/or F(ab')2	NR
da Silva & Tavares, 2012^40^	*sp.*	NR	NR		NR	NR		NR	NR	NR		NR	NR		Brazilian health ministry accredited producers	F(ab’)2	NR
Mendonça-da-Silva et al., 2017^41^	*sp.*	37 (84.1)	7 (15.9)		40 (90.9)	4 (9.1)		NR	NR	NR		NR	2.8		Brazilian health ministry accredited producers	F(ab’)2	6.0
Sachett et al., 2017^42^	*sp.*	153 (82.3)	33 (17.7)		157 (84.4)	29 (15.6)		80 (43.0)	91 (48.9)	15 (8.1)		46 (24.7)	4.9		Brazilian health ministry accredited producers	F(ab’)2	NR
Oliveira et al., 2019^43^	*sp.*	322 (78.2)	90 (21.8)		360 (87.4)	52 (12.6)		112 (27.7)	219 (54.2)	73 (18.1)		240 (58.2)	3.6		Brazilian health ministry accredited producers	F(ab’)2	NR
Resiére et al., 2020^24^	*sp.*	52 (71.2)	21 (28.8)		NR	NR		32 (42.1)	28 (36.8)	16 (21.1)		NR	9.0		Bioclon	F(ab’)2	5.0
Silva-de-Oliveira et al., 2020^44^	*sp.*	88 (88.0)	12 (12.0)		92 (92.0)	8 (8.0)		27 (27.0)	59 (59.0)	14 (14.0)		29 (29.0)	NR		Brazilian health ministry accredited producers	F(ab’)2	8.0
Heckmann et al., 2021^45^	*atrox*	34 (81.0)	8 (19.0)		40 (95.2)	2 (4.8)		0 (0.0)	33 (78.6)	9 (21.4)		NR	11.0		Bioclon	F(ab')2	3.0
Negrin et al., 2021^46^	*alternatus and pubescens*	NR	NR		NR	NR		NR	NR	NR		NR	NR		BIOL; Malbran; Brazilian health ministry accredited producers	F(ab’)2	NR
Gimenes et al., 2021^47^	*atrox*	4 (80.0)	1 (20.0)		5 (100.0)	0 (0.0)		0 (0.0)	3 (60.0)	2 (40.0)		1 (20.0)	5.6		Brazilian health ministry accredited producers	F(ab')2	v
Brasileiro-Martins et al., 2022^48^	*atrox*	110 (86.6)	17 (13.4)		115 (90.6)	12 (9.4)		17 (13.4)	72 (56.7)	38 (29.9)		43 (33.8)	7.7		Brazilian health ministry accredited producers	F(ab’)2	NR
Soares et al., 2022^49^	*sp.*	153 (82.3)	33 (17.7)		156 (83.9)	30 (16.1)		80 (43.0)	88 (47.3)	18 (9.7)		NR	NR		Brazilian health ministry accredited producers	F(ab’)2	3.4
Houcke et al., 2023^50^	*sp.*	80 (67.2)	39 (32.8)		NR	NR		60 (50.4)	31 (26.1)	28 (23.5)		NR	6.1		Bioclon	F(ab’)2	6.0
Toffano et al. 2023^51^	*sp.*	37 (78.7)	10 (21.3)		33 (70.2)	14 (29.8)		6 (16.2)	19 (51.4)	12 (32.4)		NR	6.0		Brazilian health ministry accredited producers	F(ab’)2	8.0
Coutinho et al., 2023^52^	*sp.*	206 (76.9)	62 (23.1)		NR	NR		88 (32.8)	106 (39.6)	74 (27.6)		NR	NR		Brazilian health ministry accredited producers	F(ab’)2	NR
Resiére et al., 2024^53^	*lanceolatus*	292 (75.1)	97 (24.9)		227 (58.4)	162 (41.6)		241 (70.9)	58 (17.1)	41 (12.1)		NR	4.5		Sanofi Pasteur	F(ab’)2	4.2
Pujo et al., 2025^54^	*atrox*	63 (62.4)	38 (37.6)		NR	NR		47 (46.5)	29 (28.7)	25 (24.8)		NR	14.8		Bioclon	F(ab’)2	6.0
**Total/sample size (%)^b^**	**–**	**3247/4320 (75.2)**	**1073/4320 (24.8)**		**2494/3259 (76.5)**	**765/3259 (23.5)**		**1233/3483 (35.4)**	**1553/3483 (44.6)**	**697/3483 (20.0)**		**965/2570 (37.5)**	**5.7 (0.05)^c^**		–	–	**5.5 (0.04)^c^**

F(ab')2: fragmented antibody; IgG: immunoglobulin G; NR: not reported; *sp*.: species.

^a^ Examples of interventions are tourniquet, traditional medicine and incision.

^b^ Sample size refers to the total number of participants in studies reporting the variable.

^c^ Numbers show weighted averages with standard deviations in the parentheses.

Envenomation severity ranged from mild to severe, with moderate envenomation being the most commonly reported (44.6%; 1553/3483 victims). Of the studies reporting on bite site, 76.5% (2494/3295) of the victims sustained bites to the lower limb. 

Notably, 37.5% (965/2570) of victims had received lay first-aid interventions, such as tourniquets, incisions and traditional medicine treatments, before receiving antivenom treatment. In one instance, one victim had received an injection of kerosene in the bite site.^39^ These measures may have influenced the treatment outcomes. On average, victims received antivenom treatment 5.7 hours (standard deviation, SD: 0.05) after being bitten, which is within the recommended 6-hour timeframe to prevent clinical aggravation.^50^ However, treatment times varied from 1.5 to 19 hours.

The antivenoms administered were from various manufacturers. In Brazil, studies primarily used antivenoms from its health ministry’s accredited producers (*Instituto Butantan*, *Instituto Vital Brazil* and *Fundação Ezequiel Dias*). While some studies did not specify the exact product, all antivenoms from these institutes are produced under similar conditions and are comparable.^13^ Other producers included *Instituto Clodomiro Picado* from Costa Rica; *Institut Pasteur* and Sanofi Pasteur from France; *Instituto Nacional de Salud* and Probiol from Colombia; Bioclon from Mexico; *Instituto Nacional de Higiene y Medicina Tropical Leopoldo Izquieta Pérez* from Ecuador; and BIOL and Malbran from Argentina. Except for *Instituto Clodomiro Picado *(Costa Rica)* and Instituto Nacional de Salud* (Colombia), which manufacture antivenoms composed of integral immunoglobulin G (IgG) molecules, all other antivenoms consist of antibody fragments (F(ab')2) derived from pepsin digestion of horse IgG antibodies. On average, victims received an initial dose of 5.5 (SD: 0.04) vials of antivenom, with each vial containing 10 mL. Doses ranged from 1.5 to 9.1 vials. Intravenous administration was the most common route, only one study administered antivenom via the intramuscular route.^25^

We extracted data on relevant variables related to antivenom treatment outcomes (Table 3). The least-reported symptom after antivenom treatment was local or systemic bleeding (7.9%; 123/1551), and the most common symptom was severe oedema, reported in 23.2% of victims (239/1032). Treatment-related adverse reactions occurred in about one fifth of victims: 19.6% (589/2998) experienced early reactions and 1.6% (16/992) delayed reactions. Complications such as permanent sequelae (3.3%; 50/1512), need for haemodialysis (1.5%; 17/1139) and death (0.8%; 23/3035) were less frequently reported. The median length of hospitalization reported was 4.8 days (SD: 0.06).

**Table 3:** Description of persisting symptoms and outcomes after antivenom treatment, the Region of the Americas

Study	Sample size	Persisting symptoms after antivenom, no.							Adverse reactions, no			Outcome, no.			
		Local or systemic bleeding	Coagulopathy^a^	Severe oedema	Local damage^b^	Secondary infection	Acute kidney injury		Acute	Delayed		Death	Permanent sequelae	Received haemodialysis	Mean hospitalization stay, days
Barrantes et al., 1985^25^	13	NR	8	NR	NR	NR	NR		NR	NR		NR	NR	NR	NR
Kouyoumdjian et al., 1988^26^	37	NR	NR	2	4	5	0		NR	NR		1	1	0	8.1
Kouyoumdjian et al., 1989^27^	22	1	NR	2	2	4	0		NR	NR		1	NR	NR	8.9
Cardoso et al., 1993^13^	121	35	19	88	43	NR	NR		72	10		NR	8	NR	3.5
Jorge et al., 1995^12^	170	43	29	NR	NR	NR	NR		NR	NR		NR	1	NR	NR
Otero et al., 1996^28^	39	2	14	5	2	4	8		14	NR		0	2	NR	NR
Bucher et al., 1997^29^	40	NR	NR	6	7	NR	NR		2	1		0	1	NR	5.2
Milani Junior et al., 1997^30^	29	NR	NR	NR	1	5	4		NR	NR		3	1	1	NR
Otero-Patiño et al., 1998^10^	79	3	21	1	2	15	8		32	1		1	2	2	NR
Thomas et al., 1998^31^	68	NR	NR	NR	NR	NR	NR		4	2		1	1	NR	5.2
Fan et al., 1999^32^	101	NR	NR	NR	NR	NR	NR		25	NR		NR	NR	NR	NR
Otero et al., 1999^9^	53	6	6	4	4	6	3		20	0		0	4	0	NR
Bucaretchi et al., 2001^33^	73	NR	NR	NR	13	11	1		25	0		0	3	0	NR
Otero et al., 2002^34^	39	6	10	3	11	12	15		14	NR		4	14	NR	NR
Pardal et al., 2004^15^	74	1	10	20	2	7	NR		14	NR		0	0	NR	3.9
Jorge et al., 2004^35^	251	NR	NR	NR	12	12	NR		NR	NR		NR	NR	NR	NR
Smalligan et al., 2004^11^	210	NR	84	NR	10	NR	NR		114	NR		2	NR	NR	NR
Otero et al., 2006^14^	67	NR	40	NR	NR	21	11		13	NR		NR	1	0	NR
Mise et al., 2007^36^	665	NR	NR	NR	NR	NR	1		57	NR		7	NR	NR	NR
Otero-Patiño et al., 2007^37^	53	1	1	0	NR	16	6		10	1		0	4	0	NR
Caron et al., 2009^38^	129	NR	76	NR	NR	NR	NR		38	NR		NR	NR	NR	NR
Otero-Patiño et al., 2012^39^	72	NR	11	4	1	4	6		18	1		0	4	NR	NR
da Silva & Tavares, 2012^40^	102	NR	NR	NR	NR	NR	NR		21	NR		NR	NR	NR	NR
Mendonça-da-Silva et al., 2017^41^	44	NR	0	NR	NR	NR	0		12	0		0	0	0	NR
Sachett et al., 2017^42^	186	NR	NR	NR	NR	74	NR		NR	NR		0	0	NR	NR
Oliveira et al., 2019^43^	412	15	21	NR	NR	NR	NR		NR	NR		0	NR	NR	7.0
Resiére et al., 2020^24^	83	NR	NR	35	5	23	4		17	NR		0	NR	NR	NR
Silva-de-Oliveira et al., 2020^44^	100	4	3	NR	NR	NR	NR		NR	NR		0	NR	NR	4.0
Heckmann et al., 2021^45^	42	NR	NR	NR	NR	NR	NR		11	NR		NR	NR	1	
Negrin et al., 2021^46^	49	NR	4	NR	NR	NR	NR		4	NR		NR	NR	NR	NR
Gimenes et al., 2021^47^	5	NR	NR	5	5	4	NR		NR	NR		NR	NR	NR	NR
Brasileiro-Martins et al., 2022^48^	127	NR	NR	v	NR	NR	49		NR	NR		NR	NR	2	NR
Soares et al., 2022^49^	186	NR	NR	NR	NR	NR	NR		22	NR		NR	NR	NR	NR
Houcke et al., 2023^50^	119	NR	NR	NR	NR	31	3		15	NR		NR	NR	3	7.0
Toffano et al. 2023^51^	47	NR	NR	7	NR	20	12		NR	NR		NR	NR	3	NR
Coutinho et al., 2023^52^	268	NR	NR	57	8	148	44		NR	NR		2	3	4	4.0
Resiére et al., 2024^53^	389	6	NR	NR	NR	30	NR		9	0		1	NR	NR	1.4
Pujo et al., 2025^54^	101	NR	NR	NR	NR	NR	19		6	NR		NR	NR	1	9.0
**Total/sample size (%)^c^**	**4665 (100)**	**123/1551 (7.9)**	**357/1724 (20.7)**	**239/1032 (23.2)**	**132/1495 (8.8)**	**452/1985 (22.8)**	**194/2017 (9.6)**		**589/2998 (19.6)**	**16/992 (1.6)**		**23/3035 (0.8)**	**50/1512 (3.3)**	**17/1139 (1.5)**	**4.8 (0.06)^d^**

NR: not reported.

^a^ Up to 6 hours after antivenom administration.

^b^ Such as blisters or necrosis.

^c^ Sample size refers to the total number of participants in studies reporting the variable.

^d^ Numbers show weighted average with standard deviation in the parenthesis.

## Discussion

Snakebite envenoming by *Bothrops* species remains a major public health challenge throughout the Region of the Americas, causing substantial morbidity and mortality. These snakebites typically induce severe clinical manifestations, including extensive local tissue damage, coagulopathies, haemorrhage and systemic complications. However, variations exist in the presenting symptoms depending on the offending snake species.^26^^,^^30^^,^^53^ While antivenom is the only specific treatment and has demonstrated significant lifesaving benefits,^55^ it can be less effective for some venom-induced pathologies. This systematic review evaluates these therapeutic limitations in *Bothrops* envenomation, identifying critical gaps to guide future treatment optimization.

We observed a resurgence of publications on antivenom use in *Bothrops* envenomation from 2017 onward, suggesting a renewed interest in the topic following the World Health Organization's designation of snakebite as a priority neglected tropical disease in 2017.^56^

We were unable to identify published research on *Bothrops* envenomation from high-incidence countries including the Plurinational State of Bolivia, Guyana, Nicaragua, Panama, Peru and the Bolivarian Republic of Venezuela, which report snakebite rates exceeding the regional average.^18^ The paucity of data from these countries limits a comprehensive understanding of the full spectrum of antivenom treatment for bothropic envenoming across the Region of the Americas.

The predominant profile of snakebite victims identified in our study corroborates the general understanding that men in rural areas face elevated snakebite risk.^57^^,^^58^ The reason may be due to male-dominated occupations like farming, livestock herding and resource extraction,^59^ activities often conducted without protective gear in low-income communities, reflecting persistent gender roles in these areas.^60^

The included studies only recorded 23 deaths (0.8% of 4665 victims), indicating that antivenom is useful in preventing death, as previously shown.^24^^,^^55^ However, reversing envenomation symptoms were more challenging. In our review, severe oedema, secondary infections and coagulopathies were the most frequently reported clinical complications in patients bitten by *Bothrops* snakes, about one fifth of patients experienced one of these complications. Oedema results from venom toxins and host’s inflammatory response to envenomation,^61^ and may progress to compartment syndrome requiring fasciotomy,^51^ which might lead to tissue loss or amputation. While the efficacy of fasciotomy is questioned,^62^ antivenom alone may not halt the inflammatory process, suggesting that adjunct anti-inflammatory therapies could be beneficial.

Victims who developed secondary infections, such as abscesses, cellulitis and necrotizing fasciitis, may have permanent impairment. How secondary infections following *Bothrops* snakebites develop remains incompletely understood. However, infection risk appears to correlate with envenomation severity,^63^ indicating that early venom neutralization may reduce incidence. No standardized antibiotic exists, and causative pathogens are often unidentified.^35^^,^^42^ However, resistant bacteria have been identified in the mouths of *Bothrops* snakes,^64^ which can further complicate treatment.

Coagulopathy, a hallmark of *Bothrops* envenomation, is primarily caused by venom metalloproteinases and serine proteinases toxins that degrade fibrinogen and impair platelet aggregation.^65^ Restoring coagulation is crucial to prevent bleeding, which could potentially lead to lethal complications.^43^ Therefore, enhancing antivenom potency, by boosting the concentration of specific antibodies, and using enzyme inhibitors^66^^,^^67^ may improve outcomes. We chose to report on measurements of coagulation only after 6 hours following antivenom therapy, to ensure that the outcome was attributable to the initial treatment. However, clinicians often administer additional doses of antivenom beyond this period until coagulation normalizes. These subsequent treatments were inconsistently documented across reviewed articles, making it difficult to extract precise information on interventions administered after the six-hour mark. Local and systemic bleeding, on the other hand, had a lower prevalence after antivenom therapy, corroborating that *Bothrops* haemotoxic effects can indeed be effectively counteracted by antivenom.^45^

Acute kidney injury, the leading cause of death in *Bothrops* envenomation, occurred in 10 percent of patients, and is characterized by oliguria and an increase in serum creatinine levels and may require dialysis.^68^ While its pathogenesis is unclear, coagulopathy and nephrotoxicity may play a key role,^69^ and elevated circulating venom levels correlate with acute kidney injury.^48^ Therefore, proper neutralization of circulating venom may be important to prevent acute kidney injury. Notably, we observed that studies involving *Bothrops asper* bites had a greater prevalence of acute kidney injury possibly due to toxin-specific effects. However, further investigations are needed. 

About one in 10 patients had local tissue damage, caused by synergistic toxin action and host response. Timely treatment is paramount to prevent disproportionate inflammatory damage. Evidence suggests that endogenous mediators of tissue injury may be counteracted by anti-inflammatory therapies.^70^

Our review shows that early adverse reactions are prevalent following antivenom therapy. These reactions result from the administration of large quantities of heterologous proteins, which interact with the patient’s immune system. However, most of these adverse effects were reported to be mild infusion-related reactions, such as cutaneous rash, coughs and swelling, which were effectively reversed with specific treatment. Nevertheless, life-threatening complications may occur, requiring close patient monitoring. In the included studies, late-onset serum sickness, characterized by the deposition of excessive circulating immune complexes, causing fever, headaches, polyarthritis and skin rash,^71^ was rare. This finding may reflect prompt antihistamine treatment of early reactions, but can also result from poor patient follow-up, underestimating its occurrence.^28^ Encouragingly, adverse events appear to be declining, as more of the recent articles reported fewer events, possibly reflecting improvements in antivenom production. However, despite this progress, the problem persists. While some preventive treatments for antivenom therapy have been tried, they still need validation.^72^ Furthermore, antivenom manufacturers are urged to improve their purity standards to reduce adverse effects. 

While the incidence of permanent sequelae in our analysis was low, the possible poor patient follow-up likely hinders our full understanding of the issue.^17^^,^^28^ Case reports have shown long-term sequelae following *Bothrops* snakebite injuries, such as disabilities,^73^ reduced mobility,^74^ renal failure up to 60 months after envenomation^75^ and coma due to intracranial haemorrhagic stroke.^76^ These examples emphasize the necessity for comprehensive care following envenomation. The limited documentation of long-term effects calls for further research to elucidate the role of appropriate antivenom administration in mitigating these sequelae. Moreover, psychological consequences are often underreported, yet they can represent an important sequela with lasting impact on victims’ lives. These effects are not mitigated with antivenom treatments.^77^ Both physical and psychological health problems are common in snakebite survivors, and can impair quality of life and socioeconomic stability for survivors and their families.^78^^,^^79^

The substantial heterogeneity in study designs, treatment protocols and patient demographics of included studies precluded a formal meta-analysis. Therefore, the conclusions in this review should be interpreted within this context of variability. Despite this limitation, we could still make clinically relevant observations based on conserved venom pathobiology across species and antivenoms' common mechanism of toxin neutralization via antibody binding. Across studies, some patients continued to experience symptoms after antivenom administration, indicating incomplete venom neutralization. These findings highlight critical gaps in current therapies, emphasizing the need for targeted research on refractory manifestations to inform antivenom optimization. Ideally, standardized outcome measures should be used to allow future comparisons. 

*Bothrops* envenomation is the most common snakebite in the Region of the Americas and represents a persistent public health concern. Emerging One Health challenges, such as climate change and human-driven landscape changes, are likely to exacerbate the problem.^80^^,^^81^ Climate change may shift snake distributions and behaviour, potentially altering envenomation epidemiology.^82^ Notably, recent evidence demonstrates a direct correlation between elevated temperatures, deforestation and an increase in *Bothrops* snakebite.^83^ While such ecological changes may also drive local extinctions of some venomous species,^84^ the present clinical challenges justify the need for improved therapeutic strategies, amid an unpredictable epidemiological future.

To conclude, this review underscores the challenges associated with antivenom therapy for treating *Bothrops*-related incidents. Despite advancements in research, adverse reactions following antivenom administration remain prevalent, necessitating continuous efforts to refine antivenom manufacturing processes. Furthermore, persistent clinical complications such as severe oedema, secondary infections and coagulopathy highlight the need for innovative treatment strategies, including the exploration of adjunct therapies and targeted interventions. The disparities in research coverage across affected regions emphasize the importance of global collaboration to effectively address snakebite morbidity. Moving forward, concerted efforts are required to enhance both preventive measures and care following envenomation to mitigate the long-term impact of *Bothrops* snakebites on patient health and well-being.
